# Solar ultraviolet radiation exposure, and incidence of childhood acute lymphocytic leukaemia and non-Hodgkin lymphoma in a US population-based dataset

**DOI:** 10.1038/s41416-024-02629-3

**Published:** 2024-02-29

**Authors:** Mark P. Little, Jim Z. Mai, Michelle Fang, Pavel Chernyavskiy, Victoria Kennerley, Elizabeth K. Cahoon, Myles G. Cockburn, Gerald M. Kendall, Michael G. Kimlin

**Affiliations:** 1 Radiation Epidemiology Branch, Division of Cancer Epidemiology and Genetics, National Cancer Institute, NIH, DHHS, Bethesda, MD 20892-9778 USA; 2 Faculty of Health and Life Sciences, Oxford Brookes University, Headington Campus, Oxford, OX3 0BP UK; 3 Department of Public Health Sciences, University of Virginia School of Medicine, Charlottesville, VA 22908-0717 USA; 4 Biostatistics Branch, Division of Cancer Epidemiology and Genetics, National Cancer Institute, NIH, DHHS, Bethesda, MD 20892-9778 USA; 5 Department of Preventive Medicine, Keck School of Medicine, University of Southern California, Los Angeles, CA USA; 6 Cancer Epidemiology Unit, University of Oxford, Richard Doll Building, Old Road Campus, Headington, Oxford, OX3 7LF UK; 7 Institute of Evidence Based Medicine, Bond University, Robina, Gold Coast, QLD 4226 Australia

**Keywords:** Risk factors, Epidemiology, Acute lymphocytic leukaemia, B-cell lymphoma, T-cell lymphoma

## Abstract

**Background:**

Acute lymphocytic leukaemia (ALL) and non-Hodgkin lymphoma (NHL) are among the commonest types of childhood cancer. Some previous studies suggested that elevated ultraviolet radiation (UVR) exposures increase ALL risk; many more indicate NHL risk is reduced.

**Methods:**

We assessed age<20 ALL/NHL incidence in Surveillance, Epidemiology and End Results data using AVGLO-derived UVR irradiance/cumulative radiant exposure measures, using quasi-likelihood models accounting for underdispersion, adjusted for age, sex, racial/ethnic group and other county-level socioeconomic variables.

**Results:**

There were 30,349 cases of ALL and 8062 of NHL, with significant increasing trends of ALL with UVR irradiance (relative risk (RR) = 1.200/mW/cm^2^ (95% CI 1.060, 1.359, *p* = 0.0040)), but significant decreasing trends for NHL (RR = 0.646/mW/cm^2^ (95% CI 0.512, 0.816, *p* = 0.0002)). There was a borderline-significant increasing trend of ALL with UVR cumulative radiant exposure (RR = 1.444/MJ/cm^2^ (95% CI 0.949, 2.197, *p* = 0.0865)), and significant decreasing trends for NHL (RR = 0.284/MJ/cm^2^ (95% CI 0.166, 0.485, *p* < 0.0001)). ALL and NHL trend RR is substantially increased among those aged 0–3. All-age trend RRs are most extreme (increasing for ALL, decreasing for NHL) for Hispanics for both UVR measures.

**Conclusions:**

Our more novel finding, of excess UVR-related ALL risk, is consistent with some previous studies, but is not clear-cut, and in need of replication.

## Introduction

Acute lymphocytic leukaemia (ALL) and non-Hodgkin lymphoma (NHL) are among the most common cancers in childhood in developed countries, in that order [1, 2]. The reported increasing incidence rates seen in the US and in other developed countries in pediatric lymphoid leukaemia rates [3], in particular for B-cell precursor ALL (but not for T-ALL nor acute myeloid leukaemia (AML)) [4], and NHL [3, 5] point towards a role for the modern lifestyle. To date there are relatively few well-established environmental risk factors for such paediatic cancers. For ALL these include ionising radiation [6, 7], heavy birth weight [8–11] and male sex [10]. NHL is not generally thought to be associated with ionising radiation exposure [6, 7]. There is a modest but significant socio-economic gradient for ALL, at least in the UK and USA, with increasing rates of ALL in higher socioeconomic classes [12–14]. There is also a substantial body of data suggesting a role for infections in childhood ALL [15–17]. The aetiology of NHL in childhood is poorly understood, the only well-established risk factors being those associated with dysregulation of the immune system [18]. There are known difference in rates of most common types of childhood cancer between racial/ethnic groups [19].

A Finnish cancer incidence study suggested that rates of childhood ALL slightly increased during the lighter part of the year, the increase being most pronounced for children aged 2-4 [20]. A study of childhood haematological malignancies in France demonstrated increasing rates of precursor B-cell ALL with increasing levels of solar ultraviolet radiation (UVR), but no significant variation of NHL [21]. A Greek case/control study reported significant reduction in rates of childhood NHL associated with more than 15 days annual sunbathing [22]. A meta-analysis of population-level cancer incidence data from 57 countries suggested decreased risk of ALL and increased risk of NHL in childhood associated with increased solar exposure, although only the former trend was significant (*p* < 0.01) [23].

In the current study we analyse childhood ALL and NHL risk in relation to solar exposure in the current Surveillance, Epidemiology and End Results (SEER) data [2] aggregated at the county level. We shall consider a number of different metrics of ambient solar exposure, and assess variations in solar-associated risk of ALL and NHL by sex and major racial/ethnic group.

## Materials and methods

### Study population

County level SEER22 data for cases diagnosed in 2000–2020 was used [2] in population-based SEER cancer registries, restricting to ALL and NHL cases under the age of 20 (not inclusive). We included registries pertaining to parts of the states of California, Connecticut, Georgia, Idaho, Illinois, Iowa, Kentucky, Louisiana, Massachusetts, New Jersey, New Mexico, New York, Texas, Utah and Washington; as detailed in the Supplement A (Methods) certain other states in SEER22 were omitted for various reasons. In the analytical cohort, there were a total of 1078 counties, with total populations (under age 20) ranging from 25 to 2.68 ×10^6^, with mean 3.67 ×10^4^.

ALL was defined by the lymphoid neoplasm recode 2021 Revision of ‘2(a)1 Precursor Non-Hodgkin lymphoma, B-cell’, and ‘2(b)1 Precursor Non-Hodgkin lymphoma, T-cell’ [24]. For cases diagnosed in 2000–2012, we additionally included ALL cases that had a histology code of the international classification of disease for oncology (ICD-O-3) ‘9727’ [25].

NHL was defined using ‘2(a)2 Mature Non-Hodgkin lymphoma, B-cell’, ‘2(a)3 Non-Hodgkin lymphoma, B-cell, NOS ‘, and ‘2(b)2 Mature Non-Hodgkin lymphoma, T-cell’, or with a ICD-O-3 histology code of ‘9832’ [24]. For cases diagnosed in 2013–2020, we additionally included NHL cases with a histology code of ‘9727’ [25].

We restricted analysis to the four main racial/ethnic groups, namely white non-Hispanic, black non-Hispanic, Hispanic (all races), and non-Hispanic Asian or Pacific Islanders. Further details of groups excluded are detailed in the Supplement A (Methods). The county population-year counts used in the calculation of population-years (somewhat analogous to person–years) at risk were based on the 2000 U.S. standard population (single ages to 84 – Census P25-1130). Given the known difference in childhood cancer rates between these racial/ethnic groups [19], and the geographical heterogeneity of distribution of the various racial/ethnic groups, analysis of exposure response could be potentially confounded. We therefore adjusted for racial/ethnic group in all analyses.

### Solar radiation exposure assessment

The AVerage daily total GLObal solar radiation (AVGLO) estimates that are employed are derived used the National Solar Radiation Database (NSRAD) produced by the National Renewable Energy Laboratory (NREL) under the US Department of Energy’s Resource Assessment Program. This is the largest ground-based solar measurement network in the US, containing statistical summaries computed from hourly measurement data (with some infilling for missing data) for 239 US radiation stations for the period 1961–1990, including monthly, yearly, and 30-year average global solar radiation measures, and gives estimates of ambient solar exposure cumulated over a day, measured in W hour/m^2^. We employ county-level interpolations developed by Tatalovich et al. [26] which deliver estimates of potential solar ambient irradiance (~100–3000 nm) at 1 km² resolution in the mainland US. Linkage of SEER data to this interpolated AVGLO exposure database was via the county-level Federal Information Processing System (FIPS) code. Further details, in particular details of data mislinkage (because of incompleteness in either the SEER or AVGLO data), are given in the Supplement A (Methods).

Two candidate exposure metrics are suggested a priori, namely UVR irradiance (in units of mW/cm^2^), which is proportional to UVR power density on a surface, or UVR cumulative radiant exposure (in units of MJ/cm^2^), which is proportional to *cumulative* solar UVR energy deposition on a surface. These are measures of UVR exposure recommended by the Commission Internationale de l’Eclairage (CIE) [27]. As noted in the Supplement A (Methods) AVGLO is approximately proportional to the average total solar irradiance (in units of mW/cm^2^). We shall also use the measure of cumulative radiant exposure (in units of MJ/cm^2^). The derivation of both measures is explained in more detail in the Supplement A (Methods), and is as previously employed [28–30].

### Statistical analysis

Because of marked under-dispersion, with variance generally reduced by about 10% over the Poisson-expected rates in certain race-sex subgroups for both disease endpoints, a quasi-likelihood model was used for all model fits and tests of significance [31]. The model assumes that the expected number of cases in the stratum with population-years $P{Y}_{i}$, after UVR exposure, ${H}_{i}$ (using either irradiance or cumulative radiant exposure), with various other explanatory covariates, ${X}_{i}=({X}_{ij})$, is given by:

1 $$P{Y}_{i}\exp \left[\alpha {H}_{i}+{\sum}_{j}{X}_{ij}{\beta }_{j}\right]$$

The population in each year and subgroup defined by the stratification is summed over each separate calendar year to give the population-year total $P{Y}_{i}$ for that subgroup. Model fitting is performed in R [32] using the glm function. Other variables used for adjustment were taken from a set of demographic/socioeconomic variables measured at county level. The variables measured are described in Supplement A Table A1. To avoid variables that could potentially soak up the effect of UVR exposure, we exclude any which had absolute value of the (Pearson) correlation with UVR irradiance of 0.1 or greater. In order to avoid over-parameterised models, the Akaike Information Criterion (AIC) [33, 34] was employed to select the optimal subset of descriptive variables from this set. A mixed forward-backward stepwise algorithm was used to select the set of variables minimising AIC, using R [32]. In order to test the effect of excluding those baseline variables with correlation >0.1, this restriction was relaxed, and AIC used to select the optimal subset of descriptive variables again. We also performed sensitivity analysis via model fits in which the demographic/socioeconomic variables were omitted. Profile-likelihood confidence intervals (CI) were estimated from the quasi-likelihood [31]. All statistical tests were two-sided.

## Results

Among the four main racial/ethnic groups analysed here there are 30,349 ALL cases and 8062 cases of NHL among a population with 831,424,805 population-years of follow-up (Tables 1 and 2).

**Table 1:** Cases of acute lymphocytic leukaemia (ALL) and non-Hodgkin lymphoma (NHL), and population-years of observation under the age of 20 by sex in the SEER 22-registry data, covering the years 2000–2020.

	ALL cases	NHL cases	Population-years
Male	17,446	5337	425,371,683
Female	12,903	2725	406,053,122
Total	30,349	8062	831,424,805

**Table 2:** Major risk factors of acute lymphocytic leukaemia and non-Hodgkin lymphoma, in the SEER 22-registry data^a^.

	Acute lymphocytic leukaemia			Non-Hodgkin lymphoma		
	Cases/Population-years	Incidence rate/10^5^ person/year (95% CI)	Heterogeneity *p*-value	Cases/Population-years	Incidence rate/10^5^ person/year (95% CI)	Heterogeneity *p*-value
Attained age						
0-1	2616/80,978,616	3.230 (3.117, 3.347)	<0.0001	150/80,978,616	0.185 (0.160, 0.213)	<0.0001
2-3	7570/81,002,746	9.345 (9.151, 9.542)		496/81,002,746	0.612 (0.565, 0.662)	
4-5	5251/81,427,823	6.449 (6.288, 6.612)		595/81,427,823	0.731 (0.679, 0.785)	
6-7	3139/81,505,052	3.851 (3.727, 3.978)		644/81,505,052	0.790 (0.736, 0.846)	
8-9	2374/82,184,626	2.889 (2.782, 2.998)		627/82,184,626	0.763 (0.710, 0.818)	
10-11	2001/84,125,569	2.379 (2.283, 2.477)		745/84,125,569	0.886 (0.829, 0.944)	
12-13	2128/84,600,238	2.515 (2.417, 2.616)		859/84,600,238	1.015 (0.955, 1.078)	
14-15	2053/84,659,863	2.425 (2.329, 2.524)		1175/84,659,863	1.388 (1.318, 1.461)	
16-17	1805/85,367,537	2.114 (2.025, 2.206)		1313/85,367,537	1.538 (1.464, 1.614)	
18-19	1412/85,572,735	1.650 (1.571, 1.731)		1458/85,572,735	1.704 (1.626, 1.784)	
Racial/ethnic group						
White non-Hispanic	13,976/398,795,263	3.505 (3.448, 3.562)	<0.0001	4165/398,795,263	1.044 (1.016, 1.073)	<0.0001
Black non-Hispanic	2265/113,835,569	1.990 (1.911, 2.071)		1044/113,835,569	0.917 (0.868, 0.968)	
Hispanic (all races)	12,211/261,297,462	4.673 (4.593, 4.755)		2228/261,297,462	0.853 (0.821, 0.885)	
Asian/Pacific Islander	1897/57,496,511	3.299 (3.156, 3.447)		625/57,496,511	1.087 (1.012, 1.166)	
Sex						
Female	12,903/406,053,122	3.178 (3.126, 3.230)	<0.0001	2725/406,053,122	0.671 (0.649, 0.693)	<0.0001
Male	17,446/425,371,683	4.101 (4.044, 4.159)		5337/425,371,683	1.255 (1.225, 1.284)	
Median rent ($)						
<300	63/2,019,876	3.119 (2.427, 3.932)	<0.0001	11/2,019,876	0.545 (0.298, 0.899)	<0.0001
300–399	682/21,184,048	3.219 (2.989, 3.461)		197/21,184,048	0.930 (0.815, 1.056)	
400–499	1824/52,485,570	3.475 (3.322, 3.633)		472/52,485,570	0.899 (0.826, 0.977)	
500–599	2608/79,409,342	3.284 (3.163, 3.409)		679/79,409,342	0.855 (0.797, 0.916)	
600–699	3096/88,331,217	3.505 (3.386, 3.627)		827/88,331,217	0.936 (0.878, 0.997)	
700–799	4758/128,466,209	3.704 (3.602, 3.807)		1133/128,466,209	0.882 (0.835, 0.930)	
≥800	17,318/459,528,543	3.769 (3.714, 3.824)		4743/459,528,543	1.032 (1.005, 1.060)	
Supplemental Nutrition Assistance Program (SNAP)(monthly benefit, $)						
<100	3839/97,697,897	3.929 (3.813, 4.048)	<0.0001	1045/97,697,897	1.070 (1.012, 1.129)	<0.0001
100–199	11,458/310,827,381	3.686 (3.623, 3.750)		3061/310,827,381	0.985 (0.954, 1.017)	
200–299	12,104/340,117,694	3.559 (3.499, 3.619)		3159/340,117,694	0.929 (0.900, 0.958)	
300–399	1816/52,495,236	3.459 (3.311, 3.612)		463/52,495,236	0.882 (0.812, 0.956)	
400–499	487/13,677,546	3.561 (3.270, 3.868)		141/13,677,546	1.031 (0.885, 1.192)	
500–599	334/10,131,716	3.297 (2.974, 3.642)		125/10,131,716	1.234 (1.049, 1.439)	
≥600	311/6,477,335	4.801 (4.314, 5.324)		68/6,477,335	1.050 (0.841, 1.291)	
Calendar year						
2000–2002	3964/117,103,293	3.385 (3.287, 3.485)	<0.0001	1060/117,103,293	0.905 (0.857, 0.955)	<0.0001
2003–2005	4142/118,204,624	3.504 (3.405, 3.606)		1054/118,204,624	0.892 (0.844, 0.941)	
2006–2008	4455/119,325,723	3.733 (3.631, 3.838)		1142/119,325,723	0.957 (0.908, 1.008)	
2009–2011	4523/120,369,674	3.758 (3.655, 3.862)		1083/120,369,674	0.900 (0.852, 0.949)	
2012–2014	4335/119,476,855	3.628 (3.528, 3.731)		1321/119,476,855	1.106 (1.053, 1.160)	
2015–2017	4468/119,077,144	3.752 (3.649, 3.857)		1259/119,077,144	1.057 (1.006, 1.111)	
2018–2020	4462/117,867,492	3.786 (3.682, 3.891)		1143/117,867,492	0.970 (0.920, 1.021)	
UVR cumulative radiant exposure (MJ cm^−2^)						
<0.05	2626/81,073,682	3.239 (3.124, 3.356)	<0.0001	151/81,073,682	0.186 (0.161, 0.214)	<0.0001
0.05–0.06	3071/33,731,191	9.104 (8.806, 9.409)		198/33,731,191	0.587 (0.516, 0.664)	
0.07–0.09	4835/52,767,665	9.163 (8.923, 9.407)		339/52,767,665	0.642 (0.583, 0.706)	
0.10–0.19	8743/187,479,962	4.663 (4.572, 4.756)		1434/187,479,962	0.765 (0.730, 0.801)	
0.20–0.29	4476/172,660,786	2.592 (2.522, 2.664)		1575/172,660,786	0.912 (0.872, 0.953)	
≥0.30	6598/303,711,519	2.172 (2.124, 2.222)		4365/303,711,519	1.437 (1.399, 1.476)	
UVR irradiance (mW cm^−2^)						
<0.60	850/23,840,719	3.565 (3.341, 3.799)	<0.0001	207/23,840,719	0.868 (0.766, 0.979)	<0.0001
0.60–0.64	4902/146,660,857	3.342 (3.254, 3.432)		1556/146,660,857	1.061 (1.014, 1.109)	
0.65–0.69	5548/159,269,574	3.483 (3.397, 3.572)		1731/159,269,574	1.087 (1.042, 1.133)	
0.70–0.74	2578/66,661,967	3.867 (3.727, 4.011)		577/66,661,967	0.866 (0.804, 0.931)	
0.75–0.79	4395/133,685,184	3.288 (3.196, 3.381)		1205/133,685,184	0.901 (0.856, 0.948)	
0.80–0.84	8541/215,788,030	3.958 (3.878, 4.039)		2052/215,788,030	0.951 (0.914, 0.988)	
0.85–0.89	1822/43,603,659	4.179 (3.998, 4.364)		376/43,603,659	0.862 (0.786, 0.943)	
≥0.90	1713/41,914,815	4.087 (3.905, 4.274)		358/41,914,815	0.854 (0.777, 0.936)	

^a^The univariate absolute risks are shown for a quasi-likelihood model. The 95% CI are quasi-likelihood-based. The heterogeneity *p*-values are derived from the likelihood ratio test.

For ALL there are highly significant effects of age (with risk varying in a U-shaped manner, peaking at ages 2-3 years), sex (risk for males ~1.29× females), racial/ethnic group (risks for black non-Hispanics ~0.57× white non-Hispanics, risks of Hispanics ~1.33× white non-Hispanics, risks of Asian/Pacific Islanders ~0.94× white non-Hispanics), median rent (with risk decreasing with cheaper rent), heterogeneity by SNAP and increasing trends over time (Table 2). For NHL there are highly significant effects of age (with risk increasing with age), sex (risk for males ~1.87× females), racial/ethnic group (risks for black non-Hispanics ~0.88× white non-Hispanics, risks of Hispanics ~0.82× white non-Hispanics, risks of Asian/Pacific Islanders ~1.04× white non-Hispanics), median rent (risk decreasing in lower rental-cost accommodation), heterogeneity by SNAP and increasing trends over time (Table 2). All heterogeneity *p*-values are highly significant (*p* < 0.0001).

The stepAIC algorithm suggests that the optimal background model for ALL includes age, racial/ethnic group, sex, median rent, calendar year, age × racial/ethnic group, age × sex, age × calendar year, racial/ethnic group × calendar year in that order; for NHL the optimal set of explanatory variables are age, sex, racial/ethnic group, median rent, calendar year, SNAP, age × sex, and racial/ethnic group × sex, in that order.

**Fig. 1:**
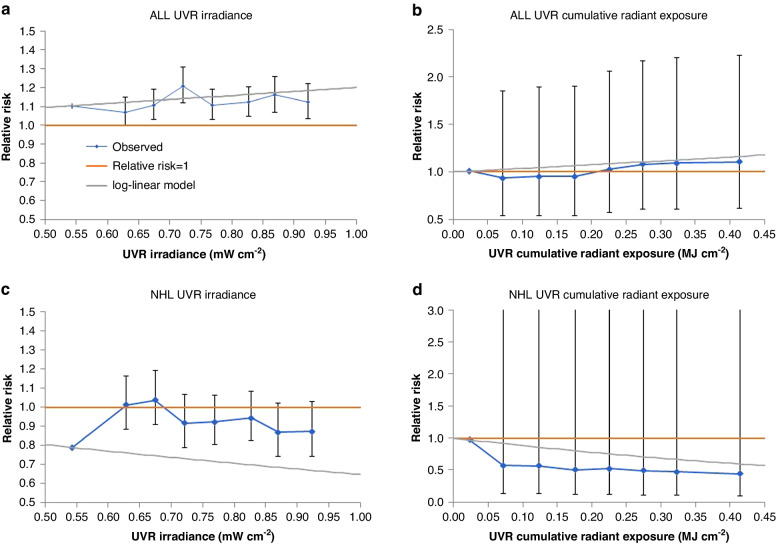
Relative risk (+95% CI) in relation to ambient solar UVR exposure. **a** acute lymphocytic leukaemia (ALL) in relation to UVR irradiance, (**b**) acute lymphocytic leukaemia (ALL) in relation to UVR cumulative radiant exposure (**c**) non-Hodgkin lymphoma in relation to UVR irradiance, (**d**) non-Hodgkin lymphoma in relation to UVR cumulative radiant exposure. Fitted models are the most complete (all racial/ethnic group, both sexes) AIC-optimal models given in Tables 3 and 4.

Table 3 and Fig. 1 demonstrate that using this model (and obvious simplifications [omitting the interaction terms in racial/ethnic group and sex] in the racial/ethnic group x sex subgroups) there is a highly significant trend of ALL with UVR irradiance, with relative risk (RR) = 1.200/mW/cm^2^ (95% CI 1.060, 1.359, *p* = 0.0040). There are increasing trends of ALL with solar exposure in various racial/ethnic group/sex subgroups, in particular in Hispanic boys (*p* = 0.0007) and girls (*p* = 0.0020) (Table 3). However, there is only a borderline significant increasing trend of ALL incidence with UVR cumulative radiant exposure, with RR = 1.444/MJ/cm^2^ (95% CI 0.949, 2.197, *p* = 0.0865), although there are significant increasing trends of ALL with UVR cumulative radiant exposure among Hispanic boys (*p* = 0.0337) and girls (*p* = 0.0133) (Table 3). Very similar results are obtained if demographic/socioeconomic variables are not used for adjustment (Table 3). Supplement A Table A2 demonstrates that there is evidence of modification of trend RR by race, for ALL (all but one *p*-value < 0.05), although very little evidence of such modification for sex (all *p*-values > 0.5). The largest UVR irradiance trend RR for ALL is for Hispanics with RR = 1.517 /mW/cm^2^ (95% CI 1.254, 1.837), compared with RR = 1.079 /mW/cm^2^ (95% CI 0.896, 1.300) for White non-Hispanics, RR = 0.903 /mW/cm^2^ (95% CI 0.537, 1.516) for Black non-Hispanics and RR = 0.708 /mW/cm^2^ (95% CI 0.442, 1.136) for Asian or Pacific Islanders (Supplement A Table A3). Likewise the largest UVR cumulative radiant exposure trend RR for ALL is for Hispanics with RR = 2.593 /MJ/cm^2^ (95% CI 1.387, 4.859), compared with RR = 0.999 /MJ/cm^2^ (95% CI 0.521, 1.913) for White non-Hispanics, RR = 1.504 /MJ/cm^2^ (95% CI 0.288, 7.772) for Black non-Hispanics and RR = 0.245 /MJ/cm^2^ (95% CI 0.047, 1.283) for Asian or Pacific Islanders (Supplement A Table A3).

**Table 3:** Trends in relative risk of acute lymphocytic leukaemia in relation to solar ultraviolet radiation exposure.

Subgroup	Cases/Population-years	Ultraviolet cumulative radiant exposure				Ultraviolet irradiance			
		Fully-adjusted		Adjusted without demographic/socioeconomic variables		Fully-adjusted		Adjusted without demographic/socioeconomic variables	
		Relative risk/MJ cm^−2^ (+95% CI)	*p*-value	Relative risk/MJ cm^−2^ (+95% CI)	*p*-value	Relative risk/mW cm^−2^ (+95% CI)	*p*-value	Relative risk/mW cm^−2^ (+95% CI)	*p*-value
White non-Hispanic, male^a^	8071/204,598,773	0.853 (0.362, 2.004)	0.7158	0.937 (0.405, 2.162)	0.8795	1.055 (0.816, 1.363)	0.6831	1.089 (0.847, 1.400)	0.5034
White non-Hispanic, female^a^	5905/194,196,490	1.336 (0.440, 4.037)	0.6089	1.430 (0.479, 4.249)	0.5214	1.119 (0.832, 1.503)	0.4580	1.142 (0.853, 1.527)	0.3721
Black non-Hispanic, male^a^	1310/57,845,212	3.991 (0.553, 28.389)	0.1695	3.510 (0.504, 24.188)	0.2042	1.447 (0.758, 2.751)	0.2619	1.373 (0.729, 2.578)	0.3260
Black non-Hispanic, female^a^	955/55,990,357	0.453 (0.041, 4.813)	0.5126	0.245 (0.025, 2.358)	0.2241	0.543 (0.266, 1.101)	0.0907	0.432 (0.219, 0.848)	0.0148
Hispanic, male^a^	6981/133,570,992	2.319 (1.067, 5.060)	0.0337	2.284 (1.074, 4.876)	0.0320	1.542 (1.201, 1.981)	0.0007	1.534 (1.203, 1.957)	0.0005
Hispanic, female^a^	5230/127,726,470	3.039 (1.260, 7.364)	0.0133	2.999 (1.264, 7.146)	0.0126	1.480 (1.154, 1.900)	0.0020	1.473 (1.154, 1.883)	0.0019
Asian/Pacific Islander, male^a^	1084/29,356,706	0.275 (0.033, 2.304)	0.2332	0.281 (0.035, 2.281)	0.2343	0.934 (0.496, 1.763)	0.8328	0.934 (0.501, 1.745)	0.8299
Asian/Pacific Islander, female^a^	813/28,139,805	0.152 (0.004, 6.027)	0.3141	0.338 (0.015, 7.739)	0.4953	0.440 (0.163, 1.192)	0.1063	0.582 (0.254, 1.340)	0.2033
Total (white + black non-Hispanic, Hispanic, Asian/ Pacific Islanders)^b^	30,349/831,424,805	1.444 (0.949, 2.197)	0.0865	1.494 (0.992, 2.249)	0.0545	1.200 (1.060, 1.359)	0.0040	1.216 (1.077, 1.372)	0.0016
						Alternative measure of irradiance used by Coste et al. [21]			
						Relative risk/100 J/cm^2^/day	*p*-value	Relative risk/100 J/cm^2^/day	*p*-value
						1.235 (1.070, 1.426)	0.0040	1.253 (1.090, 1.442)	0.0016

^a^Fully-adjusted models for age (10 group factor variable), median rent, calendar year; models without demographic/socioeconomic adjustments adjust only for age and calendar year.

^b^Fully-adjusted models for age (10 group factor variable), racial/ethnic group (4 group factor variable), sex, median rent, calendar year, age × racial/ethnic group, age × sex, age × calendar year, racial/ethnic group × calendar year; models without demographic/socioeconomic adjustments adjust only for age, racial/ethnic group, sex, calendar year, age × racial/ethnic group, age × sex, age × calendar year, racial/ethnic group × calendar year.

By contrast, Table 4 and Fig. 1 demonstrate that solar exposure appears protective for NHL, with decreases in incidence for increasing UVR irradiance with RR = 0.646 /mW/cm^2^ (95% CI 0.512, 0.816, *p* = 0.0002). This is also observed, if sometimes only at borderline levels of statistical significance, in a number of racial/ethnic/sex subgroups, in particular in white non-Hispanic boys and girls (*p* = 0.0348, *p* = 0.0282, respectively), and Hispanic boys (*p* = 0.0024) (Table 4), although not in Asian or Pacific Islander children, among whom there are indications of increased NHL risk with increasing irradiance, borderline significant for males (*p* = 0.1074) and significant (*p* = 0.0282) for females. There is also a highly significant decreasing trend of NHL with UVR cumulative radiant exposure, with RR = 0.284/MJ/cm^2^ (95% CI 0.166, 0.485, *p* < 0.0001), with again significant or borderline significant decreasing trends in white non-Hispanic boys (*p* = 0.0033) and girls (*p* = 0.0666) and in Hispanic boys (*p* = 0.0008) (Table 4). By contrast, there are indications of increased NHL incidence with increasing UVR cumulative radiant exposure among Asian or Pacific Islander children, which for girls is significant (*p* = 0.0074). Very similar results are obtained if demographic/socioeconomic variables are not used for adjustment (Table 4). Supplement A Table A2 demonstrates that there is evidence of modification of RR by race for NHL (all *p*-values < 0.05), although little evidence of such modification for sex (all *p*-values > 0.05). The lowest UVR irradiance trend RR for NHL is for Hispanics with RR = 0.545/mW/cm^2^ (95% CI 0.359, 0.829), compared with RR = 0.602 /mW/cm^2^ (95% CI 0.434, 0.833) for White non-Hispanics, RR = 0.578/mW/cm^2^ (95% CI 0.277, 1.200) for Black non-Hispanics and RR = 2.059/mW/cm^2^ (95% CI 0.933, 4.574) for Asian or Pacific Islanders (Supplement A Table A3). Likewise the lowest UVR cumulative radiant exposure trend RR for NHL is for Hispanics with RR = 0.218/MJ/cm^2^ (95% CI 0.123, 0.386), compared with RR = 0.336/MJ/cm^2^ (95% CI 0.188, 0.600) for White non-Hispanics, RR = 0.511/MJ/cm^2^ (95% CI 0.250, 1.041) for Black non-Hispanics and RR = 0.331/MJ/cm^2^ (95% CI 0.155, 0.708) for Asian or Pacific Islanders (Supplement A Table A3).

**Table 4:** Trends in relative risk of non-Hodgkin lymphoma in relation to solar ultraviolet radiation exposure.

Subgroup	Cases/Population-years	Ultraviolet cumulative radiant exposure				Ultraviolet irradiance			
		Fully-adjusted		Adjusted without demographic/socioeconomic variables		Fully-adjusted		Adjusted without demographic/socioeconomic variables	
		Relative risk/MJ cm^−2^ (+95% CI)	*p*-value	Relative risk/MJ cm^−2^ (+95% CI)	*p*-value	Relative risk/mW cm^−2^ (+95% CI)	*p*-value	Relative risk/mW cm^−2^ (+95% CI)	*p*-value
White non-Hispanic, male^a^	2836/204,598,773	0.219 (0.079, 0.604)	0.0033	0.229 (0.086, 0.609)	0.0031	0.624 (0.402, 0.967)	0.0348	0.637 (0.417, 0.971)	0.0358
White non-Hispanic, female^a^	1329/194,196,490	0.306 (0.086, 1.084)	0.0666	0.384 (0.114, 1.279)	0.1191	0.528 (0.298, 0.934)	0.0282	0.586 (0.340, 1.009)	0.0537
Black non-Hispanic, male^a^	662/57,845,212	0.200 (0.018, 2.193)	0.1884	0.110 (0.012, 1.031)	0.0533	0.437 (0.147, 1.280)	0.1314	0.331 (0.121, 0.899)	0.0301
Black non-Hispanic, female^a^	382/55,990,357	0.821 (0.020, 31.54)	0.9163	0.260 (0.012, 5.638)	0.3925	1.285 (0.247, 6.501)	0.7637	0.739 (0.191, 2.824)	0.6591
Hispanic, male^a^	1434/133,570,992	0.104 (0.028, 0.388)	0.0008	0.084 (0.025, 0.283)	<0.0001	0.425 (0.245, 0.738)	0.0024	0.386 (0.233, 0.642)	0.0003
Hispanic, female^a^	794/127,726,470	1.089 (0.280, 4.280)	0.9025	0.659 (0.193, 2.277)	0.5088	0.988 (0.555, 1.763)	0.9672	0.787 (0.468, 1.329)	0.3695
Asian/ Pacific Islander, male^a^	405/29,356,706	2.527 (0.383, 16.935)	0.3366	3.736 (0.683, 20.80)	0.1290	1.922 (0.868, 4.273)	0.1074	2.229 (1.094, 4.569)	0.0271
Asian/ Pacific Islander, female^a^	220/28,139,805	15.94 (2.092, 123.7)	0.0074	10.18 (1.626, 65.33)	0.0130	2.774 (1.115, 6.936)	0.0282	2.274 (1.004, 5.187)	0.0488
Total (white + black non-Hispanic, Hispanic, Asian/ Pacific Islanders)^b^	8062/831,424,805	0.284 (0.166, 0.485)	<0.0001	0.266 (0.160, 0.440)	<0.0001	0.646 (0.512, 0.816)	0.0002	0.624 (0.501, 0.778)	<0.0001
						Alternative measure of irradiance used by Coste et al. [21]			
						Relative risk/100 J/cm^2^/day	*p*-value	Relative risk/100 J/cm^2^/day	*p*-value
						0.603 (0.461, 0.790)	0.0002	0.580 (0.450, 0.747)	<0.0001

^a^Fully-adjusted models for age (10 group factor variable), median rent, calendar year, Supplemental Nutrition Assistance Program (SNAP); models without demographic/socioeconomic adjustments adjust only for age and calendar year.

^b^Fully-adjusted models for age (10 group factor variable), sex, racial/ethnic group (4 group factor variable), median rent, calendar year, Supplemental Nutrition Assistance Program (SNAP), age × sex, racial/ethnic group × sex; models without demographic/socioeconomic adjustments adjust only for age, sex, racial/ethnic group, calendar year, age × sex, racial/ethnic group × sex.

Table 5 demonstrates that there is no significant variation in risk of ALL in different age groups whether in relation to irradiance (*p* = 0.9087) or cumulative radiant exposure (*p* = 0.3174). Nevertheless, there is reduction in trend RR with increasing age, both in relation to irradiance or cumulative radiant exposure, with large (and significant) risks for the age group 0–3, particularly in relation to cumulative radiant exposure; so that for example for ages 0–3 RR = 1.269 /mW/cm^2^ (95% CI 1.024, 1.573) and RR = 20.58 /MJ/cm^2^ (95% CI 1.559, 272.0), both measures generally decreasing with increasing age, with in some cases trend RR < 1 for older ages. Table 5 also shows that the evidence for such heterogeneity for NHL is stronger, at least for irradiance (*p* = 0.0113), although less so for cumulative radiant exposure (*p* = 0.1844). As for ALL, it is notable that there are large (and significant) risks for NHL for the age group 0–3, in relation to both UVR metrics and particularly for cumulative radiant exposure; so that for example for ages 0–3 RR = 2.483/mW/cm^2^ (95% CI 1.129, 5.463) and RR = 21,881/MJ/cm^2^ (95% CI 1.960, >10^6^), both measures generally decreasing with increasing age, with in most cases trend RR < 1 for older ages. Very similar results are obtained if the analyses omit adjustment for the various demographic/socioeconomic variables.

**Table 5:** Variation of risk of acute lymphocytic leukaemia and non-Hodgkin lymphoma modified by age, using either UVR cumulative radiant exposure (MJ cm^−2^) or UVR irradiance (mW cm^−2^).

Attained age (years)	Cases	Ultraviolet cumulative radiant exposure				Ultraviolet irradiance			
		Fully-adjusted		Adjusted without demographic/socioeconomic variables		Fully-adjusted		Adjusted without demographic/socioeconomic variables	
		Relative risk/MJ cm^−2^ (+95% CI)	*p*-value	Relative risk/MJ cm^2^ (+ 95% CI)	*p*-value	Relative risk/mW cm^−2^ (+95% CI)	*p*-value	Relative risk/mW cm^−2^ (+95% CI)	*p*-value
Acute lymphocytic leukaemia^a^									
0–3	10,186	20.58 (1.559, 272.0)	0.3174	24.04 (1.941, 298.1)	0.2306	1.269 (1.024, 1.573)	0.9087	1.285 (1.043, 1.585)	0.8904
4-5	5251	3.324 (0.501, 22.08)		3.618 (0.571, 22.93)		1.209 (0.897, 1.630)		1.225 (0.915, 1.639)	
6-7	3139	3.572 (0.621, 20.57)		3.795 (0.689, 20.92)		1.325 (0.900, 1.950)		1.343 (0.921, 1.958)	
8-9	2374	2.627 (0.550, 12.58)		2.749 (0.598, 12.66)		1.316 (0.844, 2.053)		1.333 (0.864, 2.056)	
10-11	2001	2.510 (0.624, 10.12)		2.600 (0.669, 10.12)		1.376 (0.849, 2.233)		1.393 (0.870, 2.233)	
12-13	2128	0.959 (0.307, 3.000)		0.983 (0.324, 2.991)		0.983 (0.616, 1.569)		0.993 (0.629, 1.567)	
14-15	2053	0.887 (0.325, 2.422)		0.905 (0.340, 2.411)		0.945 (0.587, 1.520)		0.954 (0.600, 1.517)	
16-17	1805	0.947 (0.369, 2.431)		0.965 (0.385, 2.419)		0.971 (0.586, 1.610)		0.981 (0.599, 1.606)	
18-19	1412	1.223 (0.475, 3.156)		1.256 (0.499, 3.165)		1.128 (0.640, 1.992)		1.147 (0.659, 1.995)	
Non-Hodgkin lymphoma^b^									
0–3	646	21,881 (1.960, >10^6^)	0.1840	15,120 (2.234, >10^6^)	0.1283	2.483 (1.129, 5.463)	0.0113	2.400 (1.139, 5.061)	0.0047
4-5	595	0.265 (0.001, 47.79)		0.201 (0.001, 27.45)		0.812 (0.356, 1.846)		0.777 (0.357, 1.690)	
6-7	644	0.037 (0.001, 1.333)		0.030 (0.001, 0.893)		0.484 (0.219, 1.069)		0.462 (0.218, 0.978)	
8-9	627	1.946 (0.118, 31.99)		1.764 (0.124, 24.97)		1.209 (0.544, 2.683)		1.176 (0.552, 2.500)	
10-11	745	0.082 (0.010, 0.673)		0.072 (0.010, 0.533)		0.419 (0.201, 0.874)		0.402 (0.200, 0.805)	
12-13	859	0.283 (0.054, 1.487)		0.261 (0.054, 1.252)		0.597 (0.301, 1.180)		0.577 (0.302, 1.099)	
14-15	1175	0.469 (0.137, 1.603)		0.440 (0.137, 1.408)		0.699 (0.390, 1.253)		0.679 (0.391, 1.178)	
16-17	1313	0.346 (0.124, 0.967)		0.327 (0.123, 0.863)		0.567 (0.326, 0.985)		0.549 (0.325, 0.926)	
18-19	1458	0.209 (0.087, 0.501)		0.199 (0.087, 0.455)		0.392 (0.231, 0.662)		0.380 (0.231, 0.625)	

^a^Fully-adjusted models for age (10 group factor variable), median rent, calendar year; models without demographic/socioeconomic adjustments adjust only for age and calendar year.

^b^Fully-adjusted models for age (10 group factor variable), median rent, calendar year, Supplemental Nutrition Assistance Program (SNAP); models without demographic/socioeconomic adjustments adjust only for age and calendar year.

Supplement A Table A4 illustrates the effect of relaxing the restriction on baseline variables having correlation with UVR < 0.1. As can be seen, comparing also with the results in Tables 3 and 4, the effect of allowing these extra variables to be used is to generally weaken the UVR-associated trends, whether the generally positive trends for ALL, or the generally negative trends for NHL. Supplement A Table A5 demonstrates that there is no significant variation in risk of ALL in different median rent groups whether in relation to irradiance (*p* = 0.8023) or cumulative radiant exposure (*p* = 0.8974); Supplement A Table A6 shows that the evidence for such heterogeneity for NHL is scarcely stronger, whether in relation to irradiance (*p* = 0.1628) or cumulative radiant exposure (*p* = 0.1646). There is high negative correlation between solar exposure and latitude, with $\rho =-0.729$ (data not shown).

## Discussion

The present study has demonstrated a highly significant increase in risk of ALL for age <20 with increasing levels of solar UVR exposure (Table 3). However, the significance of the increasing trend is only seen in the Hispanic group. Although there is some overlap of RR and CI for irradiance and cumulative radiant exposure for the four ethnic groups, nevertheless there is significant heterogeneity by ethnic group using both UVR metrics (most *p* < 0.05), with trend RR highest for Hispanics (Supplement A Tables A2, A3). The increase is seen using UVR irradiance, and much less strongly using UVR cumulative radiant exposure. By contrast, there are highly significant decreases in risk of NHL, using either measure of UVR exposure (Table 4). Although there is some overlap of RR for irradiance and cumulative radiant exposure for the four ethnic groups, nevertheless there is significant heterogeneity by ethnic group using both UVR metrics (all *p* < 0.05), with trend RR lowest for Hispanics, and for most other racial/ethnic groups trend RR < 1 (Supplement A Tables A2 and A3). Both for ALL and NHL the risk appears to be concentrated in the youngest age group (0–3 years), and in both endpoints and all UVR metrics, but particularly for cumulative radiant exposure, the trend RR is increased in this age group (Table 5).

Our findings of increased risk of ALL with increasing solar exposure parallel those found in a large recent French ecological study of childhood cases, based on high quality ground-based UV measurements, somewhat similar to the AVGLO measurements used in the present study, and which demonstrated increasing risk of precursor B-cell ALL with elevated UVR, with RR = 1.41 (95% CI 1.13, 1.69) per 100 J/cm^2^ /day [21]. This is similar to the figure we derive of 1.235 per 100 J/cm^2^ /day (95% CI 1.070, 1.426) (Table 3). A Finnish study suggested that rates of childhood ALL slightly (but non-significantly) increased during the lighter part of the year (April-September), the increase being most pronounced, about 18% (and borderline significant) for children aged 2–4 [20]. However, it is the studies of long-term solar exposure which are more comparable with the structure of our data. A Californian study using AVGLO UVR data suggested a mild and borderline significant (*p* = 0.042) protective effect of increased UVR on early childhood (under age 5 year) ALL rates [35]. This study also showed a protective effect of UVR in children of Hispanic and Black mothers [35], again in contrast to our findings (Table 3, Supplement A Table A3). A meta-analysis of population-level cancer incidence data by registry suggested that rates of childhood ALL significantly increased (*p* < 0.01) with increasing degrees of registry latitude [23], again suggestive of a protective effect. A study of all-age leukaemia mortality data in Spain observed decreases with increasing latitude, suggestive of elevated UVR-associated risk [36].

Our findings of a protective effect of solar exposure on NHL (albeit not for ages 0–3) should be compared with those found in a French ecological study which reported a non-significant increased risk of NHL of RR = 1.26 (95% CI 0.89, 1.87) per 100 J/cm^2^ /day [21]. This is very much higher, and inconsistent with the significant negative trend we derive of RR = 0.603 per 100 J/cm^2^ /day (95% CI 0.461, 0.790) (Table 4). A meta analysis, conducted at the level of cancer registry (including 75 registries among 57 countries at all levels of economic development) reported weak and non-significant decreasing trends of childhood NHL with increasing registry latitude [23], suggesting a weak positive trend with UVR. Kim et al. [37] conducted a systematic review and meta-analysis of 17 case-control and 9 cohort studies, all but two covering the full age range, and all relating to developed countries (Australia, Denmark, Europe, France, Germany, Greece, Italy, Norway, Singapore, Sweden, UK, USA) mostly in the northern hemisphere, suggested that NHL risk decreased with increasing personal sunlight exposure in relation to a number of metrics, relating both to solar exposure in childhood and in adulthood. On the other hand, Lu et al. [38] conducted a systematic review and meta-analysis of 10 case-control and 1 cohort studies of occupational exposure, again of developed countries (Australia, Denmark, Europe, Germany, Singapore, Sweden, USA) mostly in the northern hemisphere, which suggested a weak positive association of NHL with solar exposure. Petridou et al. [22] studied 87 cases of NHL at ages 0–14 from a Greek national network of oncology units together with 164 age/sex-matched controls. Average time spent sunbathing per year was determined by interviewing guardians. There was a significant (*p* = 0.002) reduction in rate of childhood NHL incidence associated with more than 15 days annual sunbathing. The Interlymph Consortium case-control study, analysed by combining trends for each centre, each one in a developed country (Australia, Canada, Denmark, France, Germany, Ireland, Italy, Spain, Sweden, UK, USA) mostly in the northern hemisphere, using meta-analytic tools, suggested weak protective effects of UVR for NHL at ages 10–17 in relation to daily hours of UVR exposure, which were borderline significant (*p* = 0.06) in relation to total sun exposure [39]. A small Californian study using the same AVGLO assessments of UVR as employed here suggested a modest and non-statistically significant (*p* > 0.1) protective effect of increased solar UVR on childhood NHL, in particular in relation to children of Hispanic and White mothers [35]. There are also a number of all-age studies, in particular Grant [36] who documented increasing trends of all-age NHL mortality with increasing latitude in Spain, suggesting a protective effect of solar exposure. Analysis of all-age NHL incidence in the US at the level of cancer registry, with UVR assessed via data from the satellite-based Total Ozone Mapping Spectrometer (TOMS) database, documented lower incidence among those registries with higher UVR quintile for many subtypes of NHL [40]. A defect of certain of these studies [21, 23, 35] is their ecological design. In all save the French [21] and Californian [35] studies the measures of solar exposure are crude or non-existent, and many studies [36–38] are not confined to cancer in childhood. Latitude, which is used in many, is only a proxy for UVR exposure, in particular will not take account of local climatic factors such as cloud cover or height above sea level, which are also known to be important, and so these apparently conflicting findings are perhaps unsurprising.

The EUROSUN UVR data used by Coste et al. [21] is probably the strongest UVR measure apart from the AVGLO data employed here and in the study of Lombardi et al. [35]. The EUROSUN UVR data is satellite based, although with validating ground-based data, so contrasting with the purely ground-based AVGLO data used here. France spans approximately 41.6–51.0 degrees North in latitude, whereas the 15 states in our study cover the range of 25.8–49.0 degrees North, thereby providing us with a much wider variation in UVR irradiance.

Our finding of concentration of UVR risk for ALL among those aged 0–3 (Table 5) is not without precedent. The study of Coste et al. [21] also reported a significant interaction (*p* = 0.007) between UVR-associated ALL risk and age, so that only for age under 5 years was the trend significant. However, the concentration of UVR risk for NHL among those aged 0–3 (Table 5) is more novel. These findings suggest powerful factors operating soon after (or before) birth. However, like all novel findings, they require replication.

A major strength of our study is the large size, using prospectively gathered cancer status data, which is linked with an independent set of county-level solar exposure measures. The availability of a rich set of lifestyle and environmental measures, albeit ecological (measured at the level of county) is also a strength. However, we were not able to examine the UVR effect in relation to different histological subtypes of ALL/NHL with varying aetiologies. The solar exposure measurements used in our study are based on interpolated solar exposure measurements derived from a 30-year series of measurements at 215 measurement stations distributed across the contiguous 48 US states [26]. What is used here is therefore a climatic average for a region and does not take account of year-to-year variations in solar exposure. The spatial resolution, to the level of US counties is a potential limitation, although the evidence is that UVR does not vary much over even relatively large (100 km square) geographical units [41]. Solar exposure of an individual living at a specific location will exhibit much greater fluctuations than ambient variation because of differences in time spent outdoors and proximity to shade on different days throughout the year. Furthermore, the solar UVR dose absorbed by the skin (assuming that to be the relevant factor) will be further modified by the use of photoprotective agents such as hats, clothing and sunscreens. There is evidence that people tend to cover up more at lower latitudes [42], which implies that personal level exposures might be less than indicated by the ambient exposure data, suggesting a likely underestimation of the slope of the cancer-solar irradiance response. We also note that there is evidence for different use of sun protective measures amongst different racial/ethnic groups [43–45]. Our analyses (Supplement A Tables A5 and A6) demonstrate no evidence of socioeconomic modifiers of UVR risk. However, the variation of solar exposure from year to year in this measurement set is relatively slight [46] and climatic norms will give far more representative sunlight values for the region of interest. Given that disease counts and underlying estimates of populations in our database are available only for complete calendar years, this may not matter too much, but inevitably there will be inaccuracies in assessing exposure, even assuming it was known which solar exposure metric, UVR irradiance *vs* UVR cumulative radiant exposure, was the more relevant, about which information is lacking. The use of these two methods of measuring solar exposure is a novelty of our study, which has not been attempted hitherto.

It is not known what the relevant exposure period for solar exposure is likely to be for the paediatric cancers considered here. It is suspected that the relevant exposure for such cancers is very early in life, as our analysis of interaction of UVR-associated risk with age might suggest (Table 5). As such correlating with solar exposure at diagnosis, an inescapable feature of the SEER registry data is likely to be not altogether the correct thing to do. However, with paediatric cancers, and particularly ALL, most of which occur under the age of 5, one can be much more confident that the solar exposure being measured relates to the entire duration of life up to the point of development of cancer. There are some mechanistic grounds for supposing that our findings with respect to ALL can be interpreted causally. Brady et al. [47] found Signature 7 UV-related mutations were enriched among a group of aneuploid pediatric ALL cases, specifically in cases with gross chromosomal alterations, including hyperdiploid (detected in 17% of samples), near haploid (35% of samples) and iAMP21 B-ALL (46% of samples). Another possible mechanism is UV-associated immunosuppression, for which there is some evidence [48]. The interaction of ALL with the immune system is complex and many autoimmune diseases exhibit elevated risk of ALL [49]. It is also clear that ALL can inhibit the immune system [50]. The protective effects of circulating vitamin D levels on many types of cancer are reasonably well known, possibly mediated via cellular gap-junctional mechanisms [51, 52], reinforced by findings of reductions in cancer mortality following vitamin D supplementation [53] although there no effects of supplementation on cancer incidence [54, 55].

As with all other studies of solar radiation our study does not take account of population migration. In our data, solar exposure is linked to the SEER county of residence at diagnosis. The effect of this is that a proportion of the population in each area, which would be larger with increasing age, will have expected solar exposure which in the worst case, of in-migration from anywhere within the US, will correspond to the US average, so that the variation in true solar exposure (of the underlying population) between areas will be to some extent over-estimated. There will also be Berkson errors resulting from applying the group means to the individual exposures, but at least to first order the effect of these on trend estimates will again be minimal [56], although uncertainties could be underestimated [57]. That said, the great advantage of studying childhood cancers is that they occur early in life, so that the effects of population migration should not be too serious [58–60]. Indeed Bell and Belanger [59] reviewing the literature on residential mobility about the time of birth in various developed countries, noted explicitly that most mothers, when they move, stayed within the same county; there was little variation between countries in this respect. Another source of error is the determination of Hispanic origin in SEER. This employs an algorithm using the patient’s surname, so that misclassification of certain cases is possible. However, as such misclassification is unlikely to vary with degree of UVR exposure it will probably not introduce bias in assessments of cancer risk in relation to UVR exposure.

In summary, our findings of a protective effect of solar exposure on NHL (albeit not for ages 0–3) are generally supported by those of a number of previous, but generally lower quality, ecological studies. Our more novel finding, of an adverse effect of solar exposure on childhood ALL, is consistent with those of a large population-based study in France, but to some extent inconsistent with findings in a case-control study in California with little UVR variation [35] and lower quality ecological studies. However, the evidence is not entirely clear-cut and all findings are in need of replication, perhaps by using individual-level data, where solar exposures can be assigned more accurately.

### Supplementary information

Supplement A

Supplement B

## Data Availability

All data and R code used for the analysis is available via online Supplement B.
