# Supplementary material for: Solar ultraviolet radiation exposure, and incidence of childhood acute lymphocytic leukaemia and non-Hodgkin lymphoma in a US population-based dataset
Source: Br J Cancer. 2024 Feb 29;130(9):1441–52. doi: 10.1038/s41416-024-02629-3 (PMC11059281; doi:10.1038/s41416-024-02629-3)
Supplement: Supplementary file 1 — Supplement A [file 41416_2024_2629_MOESM1_ESM.docx]

**Supplement A**

**Supplemental Methods and Tables.**

**Supplemental Methods**

*Study population*

County level SEER22 data for cases diagnosed in 2000-2020 was used^1^ in population-based SEER cancer registries, restricting to ALL and NHL cases under the age of 20 (not inclusive). The SEER22 data included registries pertaining to parts of the states of California, Connecticut, Georgia, Idaho, Illinois, Iowa, Kentucky, Louisiana, Massachusetts, New Jersey, New Mexico, New York, Texas, Utah and Washington; as detailed below certain other states in SEER22 were omitted for various reasons. There are a total of 1078 counties in the analytical dataset after AVGLO linkage (as detailed below).

ALL was defined by the lymphoid neoplasm recode 2021 Revision of '2(a)1 Precursor Non-Hodgkin lymphoma, B-cell', and '2(b)1 Precursor Non-Hodgkin lymphoma, T-cell'^2^. For cases diagnosed in 2000–2012, we additionally included ALL cases that had a histology code of the international classification of disease for oncology (ICD-O-3) '9727'^3^.

NHL was defined using '2(a)2 Mature Non-Hodgkin lymphoma, B-cell', '2(a)3 Non-Hodgkin lymphoma, B-cell, NOS ', and '2(b)2 Mature Non-Hodgkin lymphoma, T-cell', or with a ICD-O-3 histology code of '9832'^2^. For cases diagnosed in 2013–2020, we additionally included NHL cases with a histology code of '9727'^3^.

*Measures of UVR exposure*

*Solar radiation exposure assessment*

The AVerage daily total GLObal solar radiation (AVGLO) estimates that are employed are derived used the National Solar Radiation Database (NSRAD) produced by the National Renewable Energy Laboratory (NREL) under the US Department of Energy’s Resource Assessment Program. This is the largest ground-based solar measurement network in the US, containing statistical summaries computed from hourly measurement data (with some infilling for missing data) for 239 US radiation stations for the period 1961-1990, including monthly, yearly, and 30-year average global solar radiation measures. We employ interpolations developed by Tatalovich *et al*^4^ which deliver estimates of potential solar ambient irradiance (~100-3000 nm) at 1 km² resolution in the mainland US. Linkage of SEER data to this interpolated AVGLO exposure database was via the county-level Federal Information Processing System (FIPS) code.

Using the standard Commission Internationale de l’Eclairage (CIE) (International Commission on Illumination) terminology^5^ we estimate the UVR (=UVA+UVB) irradiance in mW/cm^2^ from the AVGLO measure $Z$ of solar daily radiant exposure in W hour/day/m^2^ estimated by Tatalovich *et al* ^4^ via the conversion factor outlined by Little *et al*^6^, namely:

$W=\frac{Z}{24\cdot10F_{tot/UVR}}$ (A1)

where $F_{tot/UVR}$ is the ratio of total (ground-level) solar output to UVR, which we estimate as $F_{tot/UVR}\approx1/0.04=25$^7^. Coste *et al*^8^ use a closely related measure, the daily average UVR radiant exposure in J/cm^2^/day for a specified location, which is given by:

$P=\frac{24\text{ x }3600\text{ x }Z}{10000\text{ x }24\text{ x }F_{tot/UVR}}=\frac{0.36Z}{F_{tot/UVR}}$ (A2)

It will also be of interest to estimate the cumulative radiant exposure (in MJ/cm^2^) up to a specified age $a$ (in years), with lag period $t_{lag}$ given by:

$E(a)=\frac{Z(3600\text{ x }24\text{ x }365.2425\text{ x max[}a-t_{lag},0])}{24\text{ x }F_{tot/UVR}\text{ x }10^{10}}=\frac{Z(3.6\text{ x }365.2425\text{ x max[}a-t_{lag},0])}{F_{tot/UVR}\text{ x }10^{7}}$ (A3)

In Tables 2-5, Supplement A Tables A2-A6 and Figure 1 we give risks for ALL and NHL in relation to these derived quantities.

*Data restrictions*

We restricted analysis to the four main racial/ethnic groups, namely white non-Hispanic, black non-Hispanic, Hispanic (all races), and non-Hispanic Asian or Pacific Islanders. The full data that would allow us to separate Asians and Pacific Islanders in the analysis is not available for anything save a subset (2000-2014) of the data. Nevertheless it is known that of the ALL cases 1818 are Asian and 79 Pacific Islander, and of the NHL cases 597 are Asian and 28 are Pacific Islander. We excluded Alaska’s registry because Alaska was a UVR outlier and only Alaskan Natives were included, in addition to excluding non–Hispanic American Indian/Alaska Native; there were 37 cases of ALL, 12 cases of NHL in a population-year total of 986,709. The Hawaii registry cases were excluded because there is no AVGLO exposure estimate for them; there were 228 ALL cases and 70 NHL cases, in a population-year total of 6,990,378. There were 13 counties for which AVGLO database linkage could not be made, in 11 cases because the county was labelled as “unknown” (although with known state) in the SEER data, and for two because the FIPS code did not exist in the AVGLO database. These included 30 ALL and 3 NHL cases, in a population-year total of 516,897. We excluded cases with unknown age or non–Hispanic unknown race. We also excluded cases not microscopically confirmed, comprising 1.1% and 1.3% of cases with ALL and NHL, respectively. In the analysis dataset we excluded cells with 0 population-years, as not so doing would have resulted in model fitting errors; a single ALL case was thereby excluded. The county population-year counts used in the calculation of population-years (somewhat analogous to person–years) at risk were based on the 2000 U.S. standard population (single ages to 84 – Census P25-1130). Given the known difference in childhood cancer rates between these racial/ethnic groups ^9^, and the geographical heterogeneity of distribution of the various racial/ethnic groups, analysis of exposure response could be potentially confounded. We therefore adjusted for racial/ethnic group in all analyses. Measures of ethnicity and racial group are SEER derived; in particular Hispanic ethnicity is derived from the surname. It was judged that there were insufficient non-white Hispanic cases to justify division of the Hispanic population into white and non-white. For example, of 12,211 Hispanic ALL cases only 541 (4.4%) were non-white.

*Statistical Analysis*

Because of marked under-dispersion, with variance generally reduced by about 10% over the Poisson-expected rates in certain race-sex subgroups for both disease endpoints, a quasi-likelihood model was used for all model fits and tests of significance^10^. The model assumes that the expected number of cases in the stratum with population-years $PY_{i}$, after UVR exposure, $H_{i}$ (using either irradiance or cumulative radiant exposure), with various other explanatory covariates, $X_{i}=(X_{ij})$, is given by:

$PY_{i}\exp[\alpha H_{i}+\sum_{j} X_{ij}\beta_{j}]$ (A4)

The population in each year and subgroup defined by the stratification is summed over each separate calendar year to give the population-year total $PY_{i}$ for that subgroup. As described above, we also employ the units of daily radiant exposure of J/cm^2^/day used by Coste *et al*^8^. Model fitting is performed in R^11^ using the glm function. Other variables used for adjustment were taken from a set of demographic/socioeconomic variables measured at county level. The variables measured are described in Table A1, a mixture of socioeconomic variables (median rent, Supplemental Nutrition Assistance Program (SNAP)) and other areal descriptors (e.g., percentage Hispanic, percentage urban, percentage colon cancer screened). Percentage colon cancer screened is a measure of the diagnostic medical facilities available in an area, and also of the population’s awareness of such screening tools, thereby representing a measure of deprivation. To avoid variables that could potentially soak up the effect of UVR exposure, we exclude any which had absolute value of the (Pearson) correlation with UVR irradiance of 0.1 or greater. In order to avoid over-parameterised models, the Akaike Information Criterion (AIC) ^12,13^ was employed to select the optimal subset of descriptive variables from this set. A mixed forward-backward stepwise algorithm was used to select the set of variables minimising AIC, using R ^11^. In order to test the effect of excluding those baseline variables with correlation >0.1, this restriction was relaxed, and AIC used to select the optimal subset of descriptive variables again. We also performed sensitivity analysis via model fits in which the demographic/socioeconomic variables were omitted. Profile-likelihood confidence intervals (CI) were estimated from the quasi-likelihood ^10^. All statistical tests were two-sided. All R code used is given in Supplement B.

**Table A1. Variables potentially used to adjust baseline risk for acute lymphocytic leukaemia and non-Hodgkin lymphoma.**

| **Variable** | **Notes** |
| --- | --- |
| **Standard demographic variables and their interactions** | |
| Age | factor variable with 10 groups |
| Sex | factor variable with 2 groups (male, female) |
| Racial/ethnic group | factor variable with 4 groups (white non-Hispanic, black non-Hispanic, Hispanic, Asian and Pacific islanders) |
| calendar year | factor variable with 7 groups – but analyzed as continuous variable using midpoint of each interval |
| age x sex |  |
| age x racial/ethnic group |  |
| age x calendar year |  |
| sex x racial/ethnic group |  |
| sex x calendar year |  |
| Racial/ethnic group x calendar year |  |
| **Sociodemographic variables with abs(correlation with UVR) < 0.1** | |
| percentage Asian |  |
| median rent |  |
| percentage urban |  |
| Supplemental Nutrition Assistance Program (SNAP) |  |
| **Other sociodemographic variables with abs(correlation with UVR) ≥ 0.1 [not used for most analyses]** | |
| percentage colon cancer screened |  |
| percentage white |  |
| percentage black |  |
| percentage Hispanic |  |
| income per capita |  |
| low income food desert |  |
| poverty rate |  |
| food desert |  |
| percentage diabetic |  |
| percentage obese |  |

**Table A2 . Significance of modification of relative risk by racial/ethnic group and sex for acute lymphocytic leukaemia and non-Hodgkin lymphoma.**

| Model type | *p*-value for modification of relative risk by race | |  | *p*-value for modification of relative risk by sex | |
| --- | --- | --- | --- | --- | --- |
|  | Cumulative radiant exposure | Irradiance |  | Cumulative radiant exposure | Irradiance |
| Acute lymphocytic leukaemia | | | | | |
| Fully-adjusted model^a^ | 0.0293 | 0.0046 |  | 0.5545 | 0.8203 |
| Model without demographic/socioeconomic adjustments^b^ | 0.0531 | 0.0072 |  | 0.5396 | 0.8125 |
| Non-Hodgkin lymphoma | | | | | |
| Fully-adjusted model^c^ | 0.0149 | 0.0266 |  | 0.0788 | 0.2843 |
| Model without demographic/socioeconomic adjustments^d^ | 0.0099 | 0.0036 |  | 0.0641 | 0.2664 |

^a^adjusted for age (10 group factor variable), racial/ethnic group (4 group factor variable), sex, median rent, calendar year, age x racial/ethnic group, age x sex, age x calendar year, racial/ethnic group x calendar year.

^b^adjusted for age, racial/ethnic group, sex, calendar year, age x racial/ethnic group, age x sex, age x calendar year, racial/ethnic group x calendar year.

^c^adjusted for adjusted models for age, sex, racial/ethnic group, median rent, calendar year, Supplemental Nutrition Assistance Program (SNAP), age x sex, racial/ethnic group x sex.

^d^adjusted for age, sex, racial/ethnic group, calendar year, age x sex, racial/ethnic group x sex.

**Table A3. Relative risk by racial/ethnic group for acute lymphocytic leukaemia and non-Hodgkin lymphoma.**

| Racial/ethnic group | Cumulative radiant exposure |  | Irradiance |
| --- | --- | --- | --- |
|  | Relative risk / MJ cm^-2^ (+ 95% CI) |  | Relative risk / mW cm^-2^ (+ 95% CI) |
| Acute lymphocytic leukaemia^a^ | | | |
| White non-Hispanic | 0.999 (0.521, 1.913) |  | 1.079 (0.896, 1.300) |
| Black non-Hispanic | 1.504 (0.288, 7.772) |  | 0.903 (0.537, 1.516) |
| Hispanic | 2.593 (1.387, 4.859) |  | 1.517 (1.254, 1.837) |
| Asian + Pacific Islander | 0.245 (0.047, 1.283) |  | 0.708 (0.442, 1.136) |
| Non-Hodgkin lymphoma^b^ | | | |
| White non-Hispanic | 0.336 (0.188, 0.600) |  | 0.602 (0.434, 0.833) |
| Black non-Hispanic | 0.511 (0.250, 1.041) |  | 0.578 (0.277, 1.200) |
| Hispanic | 0.218 (0.123, 0.386) |  | 0.545 (0.359, 0.829) |
| Asian + Pacific Islander | 0.331 (0.155, 0.708) |  | 2.059 (0.933, 4.574) |

^a^adjusted for age (10 group factor variable), racial/ethnic group (4 group factor variable), sex, median rent, calendar year, age x racial/ethnic group, age x sex, age x calendar year, racial/ethnic group x calendar year.

^b^adjusted for adjusted models for age, sex, racial/ethnic group, median rent, calendar year, Supplemental Nutrition Assistance Program (SNAP), age x sex, racial/ethnic group x sex.

**Table A4. Trends in relative risk of acute lymphocytic leukaemia and non-Hodgkin lymphoma, using all available background variables, with no exclusions for those with UV correlations >0.1. Risks are for total (white+black non-Hispanic, Hispanic, Asian/ Pacific Islanders).** Unless otherwise stated all CI are derived from the profile likelihood.

| Endpoint | Cases / Population-years | Ultraviolet cumulative radiant exposure | |  | Ultraviolet irradiance | |
| --- | --- | --- | --- | --- | --- | --- |
|  |  | Relative risk / MJ cm^-2^ (+ 95% CI) | *p*-value |  | Relative risk / mW cm^-2^ (+ 95% CI) | *p*-value |
| Acute lymphocytic leukaemia^a^ | 30,349 / 831,424,805 | 1.069 (0.662^b^, 1.727^b^) | 0.7848 |  | 1.108 (0.951^b^, 1.291^b^) | 0.1868 |
| Non-Hodgkin lymphoma^c^ | 8062 / 831,424,805 | 0.272 (0.138, 0.534) | 0.0002 |  | 0.669 (0.490, 0.914) | 0.0114 |

^a^adjusted for age (10 group factor variable), racial/ethnic group (4 group factor variable), sex, median rent, calendar year, percentage Hispanic, percentage screened for colon cancer, age x racial/ethnic group, age x sex, age x calendar year, racial/ethnic group x calendar year.

^b^using Wald-based CI.

^c^adjusted for age (10 group factor variable), sex, income, racial/ethnic group (4 group factor variable), percentage White, calendar year, food desert, Supplemental Nutrition Assistance Program (SNAP), poverty rate, age x sex, racial/ethnic group x sex.

**Table A5. Variation of relative risk of acute lymphocytic leukaemia modified by median rent, using either UVR cumulative radiant exposure (MJ cm^-2^) or UVR irradiance (mW cm^-2^)**.

| Median rent ($) | Cases | Cumulative radiant exposure | |  | Irradiance | |
| --- | --- | --- | --- | --- | --- | --- |
|  |  | Relative risk / MJ cm^-2^ (+ 95% CI) | *p*-value heterogeneity |  | Relative risk / mW cm^-2^ (+ 95c% CI) | *p*-value heterogeneity |
| <300 | 63 | 1.037 (0.294, 3.234) | 0.8974 |  | 1.170 (0.820, 1.632) | 0.8023 |
| 300-399 | 682 | 1.724 (1.002, 2.953) |  |  | 1.196 (1.014, 1.410) |  |
| 400-499 | 1824 | 1.435 (0.895, 2.299) |  |  | 1.211 (1.050, 1.396) |  |
| 500-599 | 2608 | 1.369 (0.860, 2.178) |  |  | 1.182 (1.029, 1.359) |  |
| 600-699 | 3096 | 1.387 (0.901, 2.137) |  |  | 1.171 (1.029, 1.332) |  |
| 700-799 | 4758 | 1.480 (0.963, 2.276) |  |  | 1.228 (1.081, 1.395) |  |
| 800+ | 17318 | 1.457 (0.946, 2.243) |  |  | 1.202 (1.054, 1.372) |  |

**Table A6. Variation of relative risk of non-Hodgkin lymphoma modified by median rent, using either UVR cumulative radiant exposure (MJ cm^-2^) or UVR irradiance (mW cm^-2^)**.

| Median rent ($) | Cases | Cumulative radiant exposure | |  | Irradiance | |
| --- | --- | --- | --- | --- | --- | --- |
|  |  | Relative risk / MJ cm^-2^ (+ 95% CI) | *p*-value heterogeneity |  | Relative risk / mW cm^-2^ (+ 95% CI) | *p*-value heterogeneity |
| <300 | 11 | 0.067 (0.008, 0.382) | 0.1646 |  | 0.406 (0.176, 0.825) | 0.1628 |
| 300-399 | 197 | 0.299 (0.149, 0.596) |  |  | 0.725 (0.536, 0.978) |  |
| 400-499 | 472 | 0.254 (0.138, 0.465) |  |  | 0.663 (0.509, 0.863) |  |
| 500-599 | 679 | 0.213 (0.117, 0.386) |  |  | 0.582 (0.449, 0.755) |  |
| 600-699 | 827 | 0.297 (0.171, 0.517) |  |  | 0.672 (0.528, 0.854) |  |
| 700-799 | 1133 | 0.267 (0.154, 0.460) |  |  | 0.622 (0.490, 0.788) |  |
| 800+ | 4743 | 0.303 (0.173, 0.527) |  |  | 0.629 (0.492, 0.805) |  |

**References**

1 Surveillance Epidemiology and End Results (SEER) Program (www.seer.cancer.gov). *Surveillance, Epidemiology, and End Results (SEER) Program (www.seer.cancer.gov) SEER*Stat Database: Incidence – SEER Research Plus Limited-Field Data, 22 Registries, Nov 2022 Sub (2000-2020) - Linked To County Attributes - Total U.S., 1969-2020 Counties, National Cancer Institute, DCCPS, Surveillance Research Program, released April 2023, based on the November 2022 submission.*, Accessed 9/2023, (2023).

2 Morton, L. M., Turner, J. J., Cerhan, J. R., Linet, M. S., Treseler, P. A., Clarke, C. A., *et al.* Proposed classification of lymphoid neoplasms for epidemiologic research from the Pathology Working Group of the International Lymphoma Epidemiology Consortium (InterLymph). *Blood* **110**, 695-708 (2007).

3 Dores, G. M., Devesa, S. S., Curtis, R. E., Linet, M. S. & Morton, L. M. Acute leukemia incidence and patient survival among children and adults in the United States, 2001-2007. *Blood* **119**, 34-43 (2012).

4 Tatalovich, Z., Wilson, J. P. & Cockburn, M. A comparison of Thiessen polygon, kriging, and spline models of potential UV exposure. *Cartogr Geograph Information Sci.* **33**, 217-231 (2006).

5 Sliney, D. H. & International Commission on Illumination (CIE). Radiometric quantities and units used in photobiology and photochemistry: recommendations of the Commission Internationale de L'Eclairage (International Commission on Illumination). *Photochem Photobiol.* **83**, 425-432 (2007).

6 Little, M. P., Tatalovich, Z., Linet, M. S., Fang, M., Kendall, G. M. & Kimlin, M. G. Improving assessment of lifetime solar ultraviolet radiation exposure in epidemiologic studies: comparison of ultraviolet exposure assessment methods in a nationwide U.S. occupational cohort. *Photochem Photobiol.* **94**, 1297-1307 (2018).

7 Wikipedia. *Sunlight https://en.wikipedia.org/wiki/Sunlight*, https://en.wikipedia.org/wiki/Sunlight Accessed 3/2018, (2018).

8 Coste, A., Goujon, S., Boniol, M., Marquant, F., Faure, L., Doré, J.-F., *et al.* Residential exposure to solar ultraviolet radiation and incidence of childhood hematological malignancies in France. *Cancer Causes Control* **26**, 1339-1349 (2015).

9 Chow, E. J., Puumala, S. E., Mueller, B. A., Carozza, S. E., Fox, E. E., Horel, S., *et al.* Childhood cancer in relation to parental race and ethnicity: a 5-state pooled analysis. *Cancer* **116**, 3045-3053 (2010).

10 McCullagh, P. & Nelder, J. A. *Generalized linear models. 2nd edition*. 1-526 (Chapman and Hall/CRC: Boca Raton, FL, 1989).

11 R: A language and environment for statistical computing. version 3.6.1 https://www.r-project.org (R Foundation for Statistical Computing, Vienna, Austria, 2019).

12 Akaike, H. Information theory and an extension of the maximum likelihood principle. In: B.N. Petrov & F. Czáki (eds). *2nd International Symposium on Information Theory.* 267-281 (Akadémiai Kiadó: Budapest, 1973).

13 Akaike, H. Likelihood of a model and information criteria. *J. Econometrics* **16**, 3-14 (1981).
